# Supplementary material for: Cervicovaginal microbiota and local immune response modulate the risk of spontaneous preterm delivery
Source: Nat Commun. 2019 Mar 21;10:1305. doi: 10.1038/s41467-019-09285-9 (PMC6428888; doi:10.1038/s41467-019-09285-9)
Supplement: Supplementary file 1 — Supplementary Information [file 41467_2019_9285_MOESM1_ESM.pdf]

Supplementary Materials

Cervicovaginal microbiota and local immune response modulate the risk of spontaneous preterm delivery

Michal A. Elovitz[1*], Pawel Gajer[2], Valerie Riis[1], Amy G. Brown[1], Michael S. Humphrys[2], Johanna B. Holm[2], Jacques Ravel[2*].

[1]Maternal and Child Health Research Center, Department of Obstetrics and Gynecology, Perelman School of Medicine, University of Pennsylvania, Philadelphia, PA 19104, USA

[2]Institute for Genome Sciences and Department of Microbiology and Immunology, Am, Baltimore, MD 21201, USA

Correspondence and should be addressed to M.A.E.(email: melovitz@obgyn.upenn.edu) and J.R. (email: jravel@som.umaryland.edu)

Contains:

Supplementary Figures 1-7
Supplementary Tables 1-6

Supplementary Figures

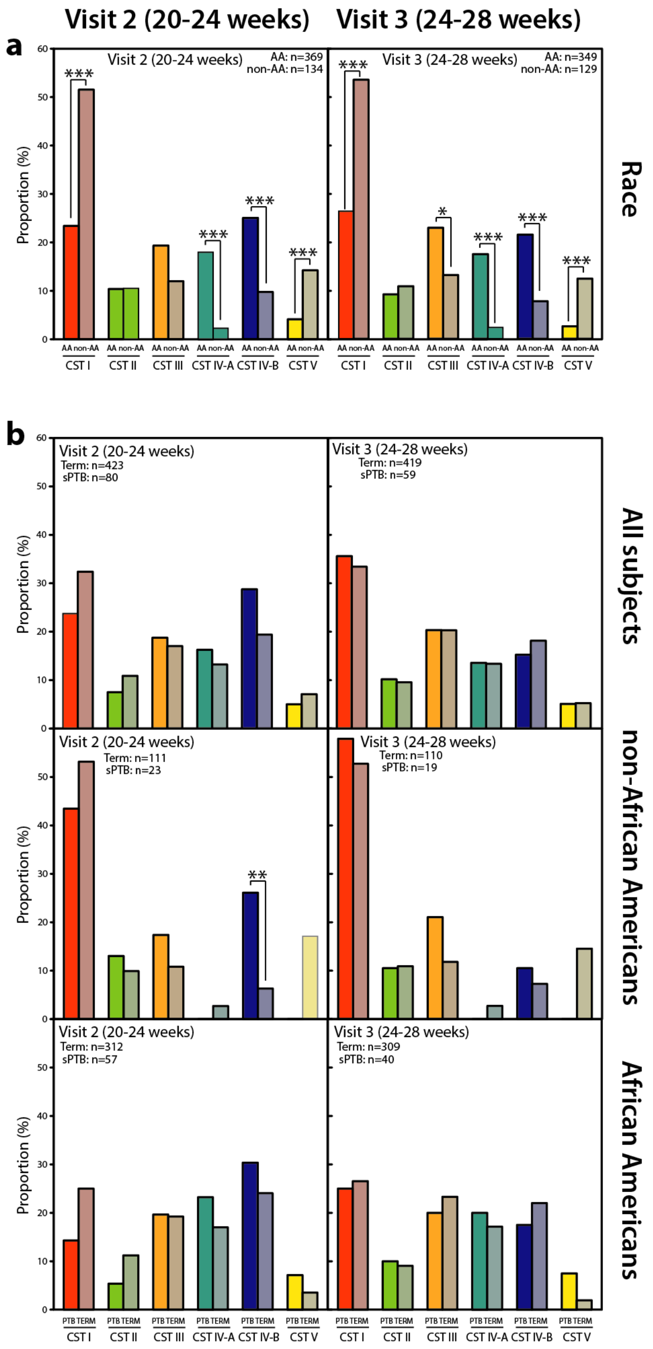

Supplementary Figure 1 | Community state types association with race and birth outcomes. (**a**) Frequency of each CST in non-AA and AA pregnant women when considering samples collected at visit 2 (20-24 weeks of gestation) or visit 3 (24-28 weeks of gestation). (**b**) Frequency of each CST stratified by outcomes (sPTB vs Term) in all subjects, non-AA and AA when considering samples collected at visit 2 or visit 3. n represents the number of samples included in the analysis. p-values were estimated using mixed effects Poisson regression models (all visits) or ordinary logistic regression models (single visit) Statistical significance is shown as * p-value <0.01, ** p-value <0.001 and *** p-value<0.0001.

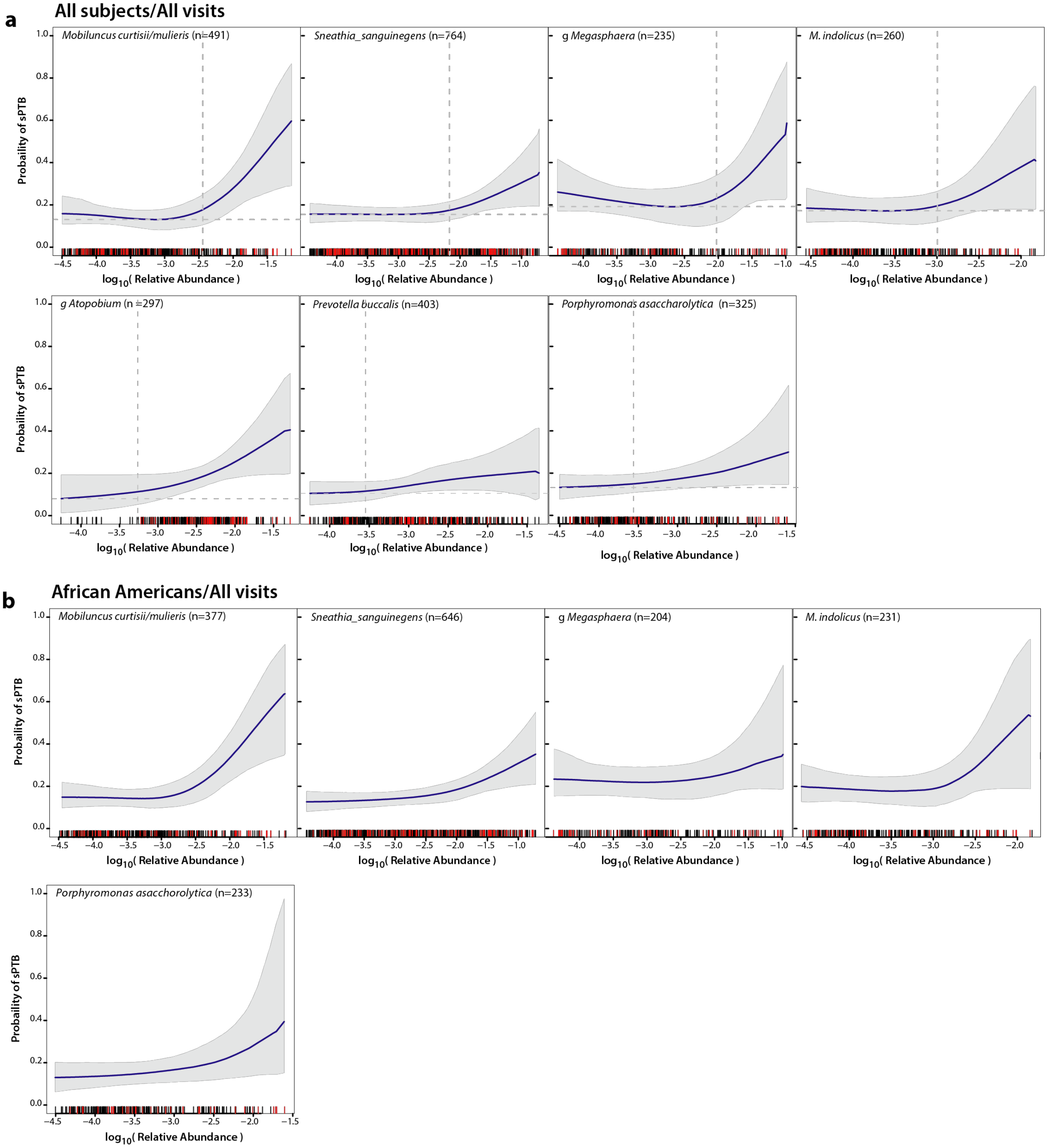
a
All subjects/All visits
Mobiluncus curtisii/mulieris (n=491)
Sneathia_sanguinegens (n=764)
g Megasphaera (n=235)
M. indolicus (n=260)
g Atopobium (n=297)
Prevotella buccalis (n=403)
Porphyromonas asaccharolytica (n=325)
Probaility of sPTB
log10(Relative Abundance)
b
African Americans/All visits
Mobiluncus curtisii/mulieris (n=377)
Sneathia_sanguinegens (n=646)
g Megasphaera (n=204)
M. indolicus (n=231)
Porphyromonas asacchorolytica (n=233)
Probaility of sPTB
log10(Relative Abundance)

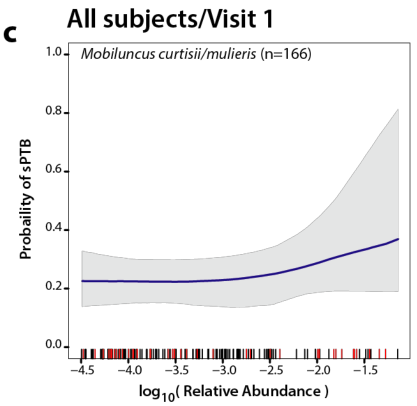

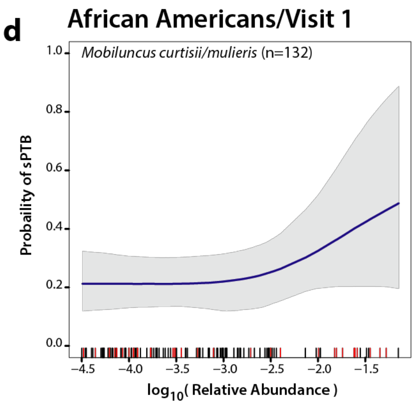

VISIT 2

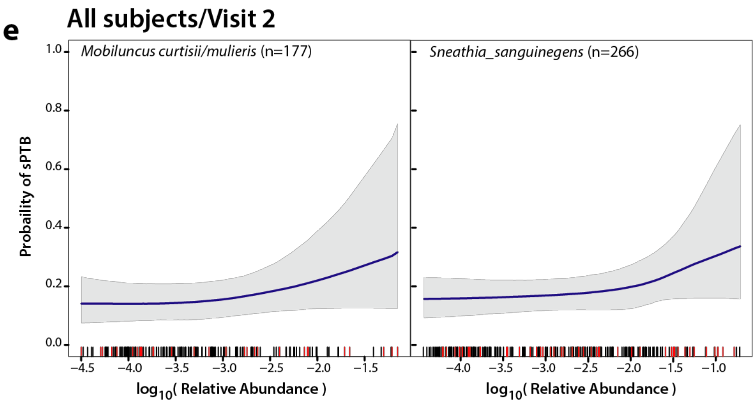

Supplementary Figure 2 | Dependence of the risk of spontaneous preterm delivery on bacterial taxa relative abundance. Dependence of the risk of sPTB (defined as <37 weeks of gestation) on the relative abundance of bacterial taxa for which the dependence was statistically significant in (**a**) all subjects/all visits, (**b**) AA/all visits, (**c**) all subjects/visit 1, (**d**) AA/visit 1, (**e**) all subjects/visit 2. n represents the number of samples in which the bacterial taxa was detected and included in the analysis. Statistically significant taxa were identified using a Bayesian logistic regression nonparametric adaptive spline models.

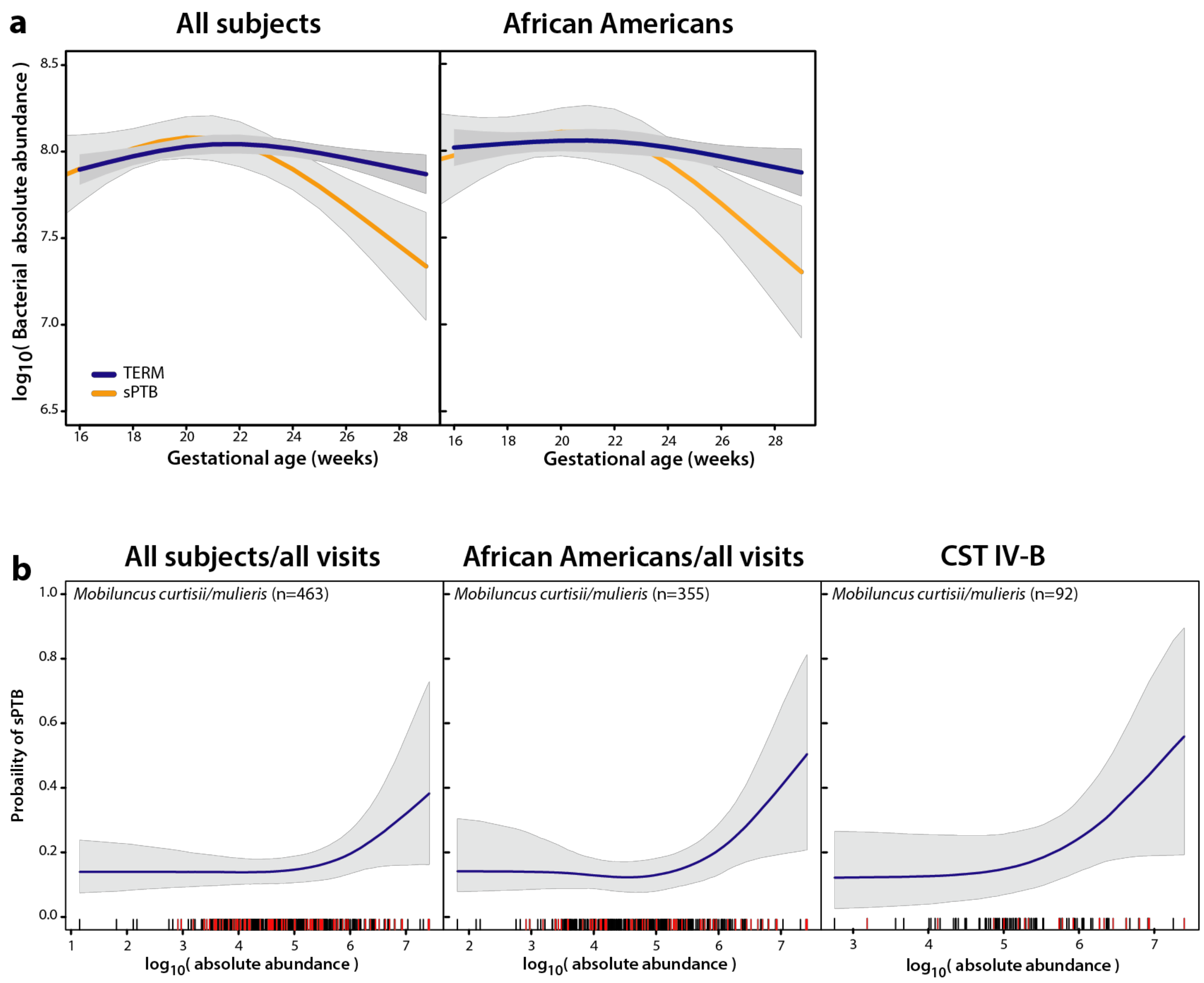

Supplementary Figure 3 | Dependence of the risk of spontaneous preterm delivery on bacterial taxa absolute abundance. (**a**) Total bacterial absolute abundance as determined by 16S rRNA gene copy numbers over gestational age in term and preterm pregnancies in all subjects and AA. Dependence of the risk of sPTB on the absolute abundance of *M. curtisii/mulieris* in (**b**) all subjects, AA and all subjects with CST IV-B vaginal microbiota. n represents the number of samples where *M. curtisii/mulieris* was detected in each group.

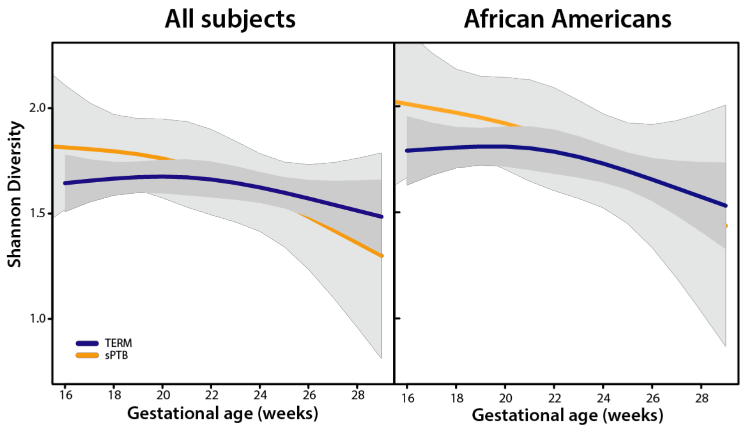

Supplementary Figure 4 | Vaginal microbiota Shannon diversity over gestational age in term and preterm pregnancies in all subjects and African American women.

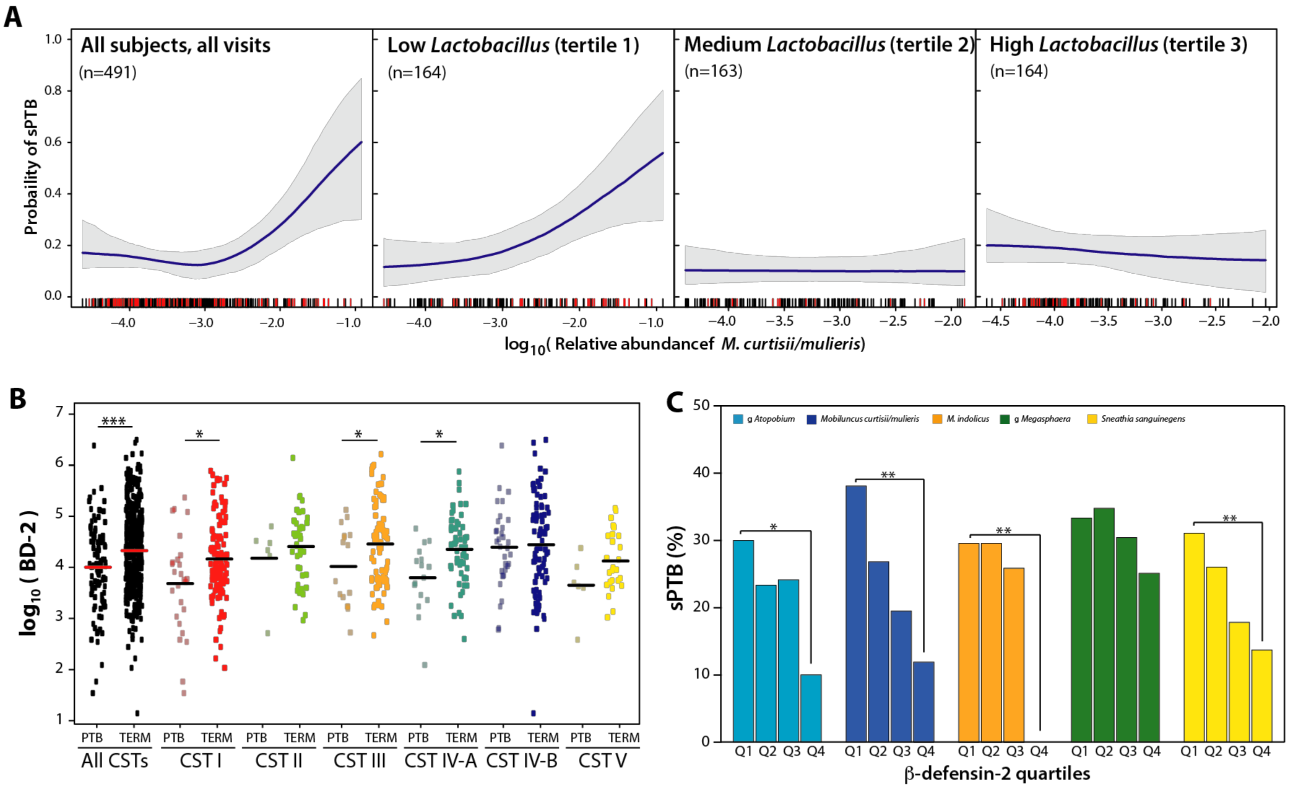

Supplementary Figure 5 | β-defensin-2 and microbiota modulate the risk of spontaneous preterm delivery. (**a**) Modulation of the risk of sPTB by relative abundance of *M. curtisii/mulieris* stratified by *Lactobacillus* spp. relative abundance tertiles within all women when all visits are considered. n represents the number of samples where *M. curtisii/mulieris* was detected. (**b**) $\log_{10}$ β-defensin-2 abundances at visit 1 in all women stratified by pregnancy outcomes and vaginal community state types. p-values estimated using a t-test. (**c**) At visit 1 in all women, the risk of sPTB associated with the relative abundance of five bacterial taxa is modulated by the abundance of β-defensin-2. p-values were estimated using a Bayesian 2-proportions binomial model with uniform prior implemented in *rstan* R package. Statistical significance is shown as * p-value <0.01, ** p-value <0.001 and *** p-value<0.0001.

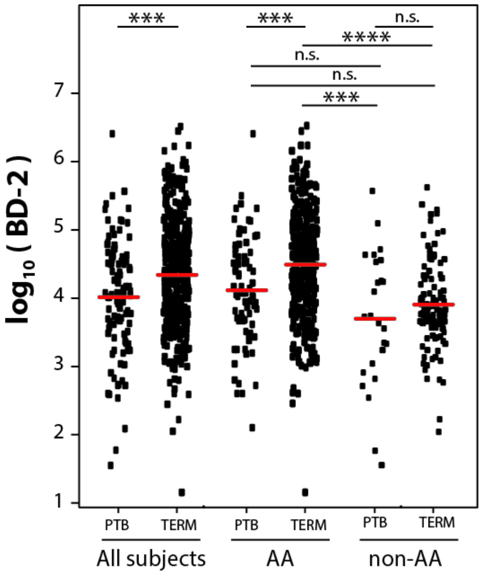

Supplementary Figure 6 | β-defensin-2, ethnicity and pregnancy outcomes. β-defensin-2 at visit 1 in AA women and pregnancy outcomes stratified by vaginal community state types. p-values estimated using a t-test. Statistical significance is shown as * p-value <0.01, ** p-value <0.001 and *** p-value<0.0001.

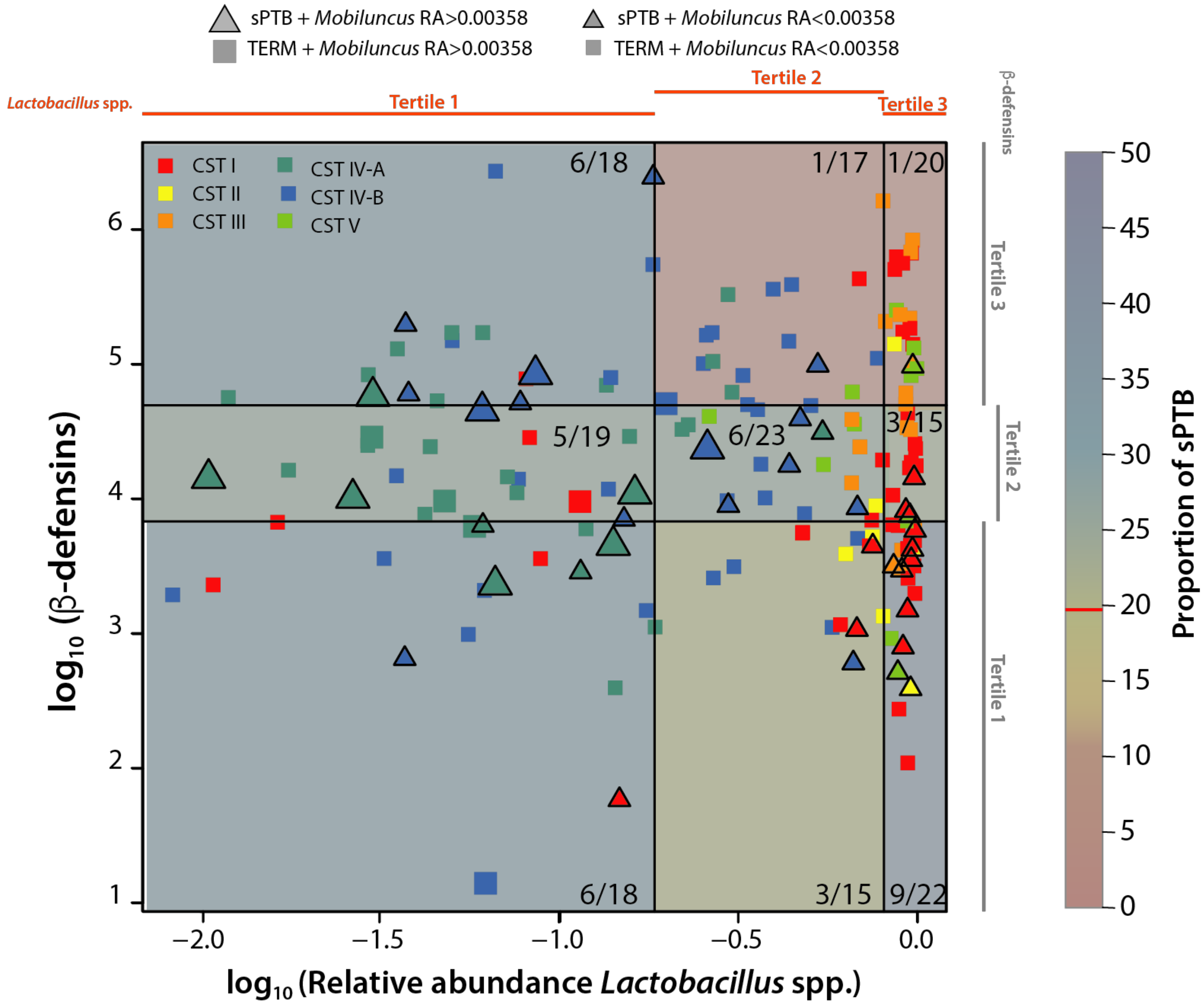

Supplementary Figure 7 | Interactions between β-defensin-2, *Lactobacillus* spp. relative abundance, *M. curtisii/mulieris* relative abundance, vaginal community state types and pregnancy outcomes in all women at visit 1. Each woman who went on to deliver preterm is represented by a triangle, while those who delivered at term by a square. Large and small triangles or squares are colored by CSTs and indicate relative abundances of *M. curtisii/mulieris* above and below its threshold value, respectively, as defined in Fig. 1. β-defensin-2 concentrations and *Lactobacillus* spp. relative abundances were stratified into tertiles. The color of each quadrant indicates the proportion of sPTBs (number of sPTB/total births).

Supplementary Tables

Supplementary Table 1. M&M study visit characteristics

Visit characteristics	Visit 1			Visit 2			Visit 3		
	Term (n=419)	sPTB (n=101)	p-value	Term (n=423)	sPTB (n=80)	p-value	Term (n=419)	sPTB (n=59)	p-value
Intercourse ≤ 24 hours	75 (17.9)	19 (18.8)	0.88	77 (18.2)	15 (18.7)	0.88	60 (14.4)	11 (18.6)	0.43
Douching ≤ 24 hours	8 (1.9)	1 (1.0)	0.99	9 (2.1)	3 (3.7)	0.42	10 (2.4)	1 (1.7)	0.99
Vaginal exam prior to visit	8 (1.9)	1 (1.0)	0.99	11 (2.6)	0	0.23	1 (0.2)	0	0.99
Antibiotics ≤ 4 weeks	22 (5.2)	5 (4.9)	0.99	17 (4.0)	3 (3.7)	0.99	10 (2.4)	2 (3.4)	0.65
Tobacco ≤ 4 weeks	22 (5.2)	8 (7.9)	0.34	16 (3.8)	5 (6.2)	0.36	16 (3.8)	4 (6.8)	0.29
Vaginal pH >=5.0	290 (69.7)	76 (76)	0.22	277 (67.4)	59 (74.7)	0.23	291 (71.7)	42 (73.7)	0.87
BMI- kg/m^2, median (IQR)	28.0 (24.1-33.2)	28.4 (23.7-34.7)	0.45	28.8 (25.0-34.1)	27.9 (24.8-33.0)	0.64	29.9 (26.0-35.0)	28.8 (25.3-33.0)	0.39

Supplementary Table 2.

Frequencies of CST per visit for all subjects, non-African Americans, African American and for each race represented in the cohort. p-values were estimated using ordinary logistic regression models. Significant p-values and trends are highlighted in bold

	Visit 1			Visit 2			Visit 3			All visits		
**All subjects**	**sPTB**	**TERM**	**p-value**	**sPTB**	**TERM**	**p-value**	**sPTB**	**TERM**	**p-value**	**sPTB**	**TERM**	**p-value**
**I**	25.2	28.7	0.479	23.8	32.4	0.128	35.6	33.4	0.74	27.3	31.5	0.34
**II**	5.83	9.98	0.196	7.5	10.9	0.366	10.2	9.55	0.879	7.44	10.1	0.737
**III**	15.5	20	0.308	18.8	17	0.708	20.3	20.3	0.992	17.8	19.1	0.805
**IV-A**	15.5	14.3	0.741	16.2	13.2	0.474	13.6	13.4	0.967	26.9	19.5	**0.0512**
**IV-B**	32	20.9	**0.0171**	28.8	19.4	**0.0607**	15.3	18.1	0.588	15.3	13.6	0.507
**V**	5.83	6.18	0.894	5	7.09	0.497	5.08	5.25	0.957	5.37	6.18	0.94
**NonAA**	**sPTB**	**TERM**	**p-value**	**sPTB**	**TERM**	**p-value**	**sPTB**	**TERM**	**p-value**	**sPTB**	**TERM**	**p-value**
**I**	44	52.8	0.43	43.5	53.2	0.4	57.9	52.7	0.677	47.8	52.9	0.444
**II**	12	9.26	0.678	13	9.91	0.656	10.5	10.9	0.96	11.9	10	0.64
**III**	16	13	0.69	17.4	10.8	0.38	21.1	11.8	0.279	17.9	11.9	0.181
**IV-A**	0	2.78	0.994	0	2.7	0.994	0	2.73	0.995	0	2.74	0.99
**IV-B**	24	6.48	**0.0129**	26.1	6.31	**0.00703**	10.5	7.27	0.626	20.9	6.69	**0.000467**
**V**	4	15.7	0.155	0	17.1	0.99	0	14.5	0.991	1.49	15.8	**0.0135**
**AA**	**sPTB**	**TERM**	**p-value**	**sPTB**	**TERM**	**p-value**	**sPTB**	**TERM**	**p-value**	**sPTB**	**TERM**	**p-value**
**I**	19.5	20.4	0.85	14.3	25	0.0859	25	26.5	0.836	19.1	24	0.161
**II**	3.9	10.2	0.0945	5.36	11.2	0.195	10	9.06	0.847	5.78	10.2	**0.0742**
**III**	14.3	22.4	0.121	19.6	19.2	0.943	20	23.3	0.641	17.3	21.6	0.204
**IV-A**	20.8	18.2	0.605	23.2	17	0.266	20	17.2	0.656	21.4	17.5	0.217
**IV-B**	35.1	25.9	0.108	30.4	24	0.316	17.5	22	0.515	29.5	24	0.125
**V**	6.49	2.88	0.137	7.14	3.53	0.217	7.5	1.94	**0.0529**	6.94	2.78	**0.00776**
**Race**	**AA**	**nonAA**	**p-value**	**AA**	**nonAA**	**p-value**	**AA**	**nonAA**	**p-value**	**AA**	**nonAA**	**p-value**
**I**	20.3	51.1	**4.05E-11**	23.4	51.5	**4.19E-09**	26.4	53.5	**5.15E-08**	23.2	52	**3.65E-25**
**II**	8.97	9.77	0.783	10.3	10.4	0.968	9.17	10.9	0.58	9.49	10.4	0.617
**III**	20.8	13.5	**0.068**	19.3	11.9	**0.0565**	22.9	13.2	**0.0203**	21	12.9	**0.000476**
**IV-A**	18.7	2.26	**0.00012**	17.9	2.24	**0.000168**	17.5	2.33	**0.000276**	18.1	2.27	**8.08E-11**
**IV-B**	27.7	9.77	**5.51E-05**	25	9.7	**0.000335**	21.5	7.75	**0.000853**	24.8	9.09	**2.07E-10**
**V**	3.59	13.5	**0.000113**	4.08	14.2	**0.000174**	2.58	12.4	**9.84E-05**	3.43	13.4	**3.22E-11**

Supplementary Table 3.

Maximum change of risk of sPTB in different sub-samples of the cohort for phylotypes relative abundance with significantly non-zero effect size (q-value significance set at 0.05) and an effect size amplitude of at least 10%. RA is relative abundance. n(TERM) and n(SPTB): n represents the number of samples and (TERM/sPTB) the number of participants included in the analysis.

Conditions	Phylotypes	Effect size	p-value	q-value	log10(RA) at mode	n(TERM)	n(sPTB)	RA(%) at mode	RA(%) Threshold (p=0.1)
**All visits and all subjects**	*Sneathia sanguinegens*	0.1986	1.00E-16	3.600E-15	3.885	629 (318)	135 (76)	0.0130	**0.00665**
	Mobiluncus curtisii/mulieri	0.4663	1.00E-16	3.600E-15	3.954	407 (240)	84 (56)	0.0111	**0.00358**
	g *Megasphaera*	0.3962	1.85E-10	4.434E-09	2.940	178 (122)	57 (41)	0.1148	**0.00949**
	M. indolicus	0.2429	1.01E-08	1.826E-07	4.125	208 (138)	52 (38)	0.0075	**0.00100**
	Porphyromonas asaccharo	0.1673	7.38E-05	1.063E-03	3.670	271 (167)	54 (40)	0.0214	**0.00054**
	g Atopobium	0.3251	2.36E-03	1.885E-02	2.400	241 (130)	56 (33)	0.3980	**0.00026**
	Prevotella buccalis	0.1042	6.10E-03	3.991E-02	3.197	347 (192)	56 (37)	0.0636	**0.00028**
**Visit V1 and all subjects**	*Mobiluncus curtisii/mulieri*	0.1460	5.80E-04	2.955E-02	4.014	126	40	0.0097	
**Visit V2 and all subjects**	*Sneathia sanguinegens*	0.1799	6.42E-05	2.528E-03	3.868	217	49	0.0136	
	Mobiluncus curtisii/mulieri	0.1762	1.01E-04	2.528E-03	3.888	146	31	0.0129	
**All visits in African Americans**	*Mobiluncus curtisii/mulieri*	0.5291	5.47E-05	1.012E-03	3.989	311 (183)	66 (45)	0.0103	
	M. indolicus	0.3607	1.06E-13	7.854E-12	3.955	182 (116)	49 (35)	0.0111	
	Sneathia sanguinegens	0.2255	1.65E-10	6.096E-09	2.677	538 (257)	108 (57)	0.2104	
	Porphyromonas asaccharo	0.2651	1.83E-06	4.501E-05	3.653	193 (121)	40 (30)	0.0222	
	g *Megasphaera*	0.1325	1.22E-03	1.802E-02	2.952	155 (104)	49 (34)	0.1117	
**Visit V1 in African Americans**	*Mobiluncus curtisii/mulieri*	0.2759	3.83E-07	2.028E-05	4.010	100	32	0.0098	
**All visits Non-African Americans**	*Lactobacillus iners*	0.1516	8.12E-04	2.719E-02	2.546	319 (112)	67(27)	0.2844	
	Atopobium vaginae	0.1122	1.44E-03	3.204E-02	3.556	214 (100)	58 (27)	0.0278	
**Visit V1 in non-African Americans**	None	n/a	n/a	n/a	n/a	n/a	n/a	n/a	
**Nulliparous women**	g *Megasphaera*	0.6796	7.95E-13	4.270E-11	3.806	67 (510)	17 (12)	0.0156	
	Sneathia sanguinegens	0.2474	1.20E-12	4.270E-11	3.889	257 (130	49 (28)	0.0129	
	Atopobium vaginae	0.1159	1.11E-04	2.632E-03	3.532	441 (173)	75 (36)	0.0294	
	g Atopobium	0.5151	1.19E-03	2.106E-02	2.509	93 (49)	19 (11	0.3097	
	Globicatella sp	0.1901	1.79E-03	2.537E-02	3.899	120 (71)	20 (13	0.0126	
	Streptococcus agalactiae	0.1706	2.55E-03	3.019E-02	4.013	86 (69)	21 (16)	0.0097	
**Multiparous women**	*Mobiluncus curtisii/mulieri*	0.4445	1.00E-16	3.600E-15	3.903	209 (133)	57 (38)	0.0125	
**CST I women**	*Lactobacillus iners*	0.2307	4.56E-06	3.149E-04	2.454	388 (156)	361 (150)	0.3516	
	Lactobacillus jensenii	0.1061	1.17E-03	4.041E-02	2.122	361 (150)	57 (29)	0.7551	
**CST III women**	None	n/a	n/a	n/a	n/a	n/a	n/a	n/a	
**CST IV-A women**	None	n/a	n/a	n/a	n/a	n/a	n/a	n/a	
**CST IVB women**	*Mobiluncus curtisii/mulieri*	0.6897	7.78E-08	2.022E-06	3.258	76 (47)	23 (16)	0.0552	
	Mycoplasma hominis	0.3466	5.08E-04	8.809E-03	3.148	98 (55)	22 (15)	0.0711	
	Sneathia sanguinegens	0.3215	1.08E-03	1.407E-02	2.737	163 (78)	36 (20)	0.1832	
**All subjects per *Lactobacillus* Tertile**	*Mobiluncus curtisii/mulieri*	0.4485	1.25E-05	4.996E-05	3.216	131 (91)	33 (29)	0.0608	
**All subjects per *Lactobacillus* Tertile**	*Mobiluncus curtisii/mulieri*	0.0037	4.52E-01	4.522E-01	3.204	139 (106)	24 (23)	0.0625	
**All subjects per *Lactobacillus* Tertile**	*Mobiluncus curtisii/mulieri*	-0.0625	2.27E-01	3.023E-01	4.017	137 (107)	27 (27)	0.0096	
**All visits in African Americans per *Lactobacillus* Tertile [illegible]**	*Mobiluncus curtisii/mulieri*	0.5732	5.12E-06	2.048E-05	3.102	95 (63)	31 (20)	0.0791	
**All visits in African Americans per *Lactobacillus* Tertile [illegible]**	*Mobiluncus curtisii/mulieri*	0.0361	3.02E-01	3.548E-01	3.140	109 (78)	16 (14)	0.0724	
**All visits in African Americans per *Lactobacillus* Tertile [illegible]**	*Mobiluncus curtisii/mulieri*	-0.0201	3.55E-01	3.548E-01	4.066	107 (83)	19 (17)	0.0086	

Supplementary Table 4.

Maxmum change of risk of sPTB in different sub-samples of the cohort for phylotypes absolute abundances with significantly non-zero effect size (q-value significance set at 0.05) and an effect size amplitude of at least 10%. RA is relative abundance. n(TERM) and n(SPTB): n represents the number of samples and (TERM/sPTB) the number of participants included in the analysis.

Conditions	Phylotypes	Effect size	p-value	q-value	log10(RA) at mode	n(TERM)	n(sPTB)	RA(%) at mode
**All visits and all subjects**	*Mobiluncus curtisii/mulieris*	0.2595	1.00E-16	3.60E-15	3.957	395 (234)	76 (51)	0.0110
**Visit 1 and all subjects**	None	n/a	n/a	n/a	n/a	n/a	n/a	n/a
**Visit 2 and all subjects**	None	n/a	n/a	n/a	n/a	n/a	n/a	n/a
**All visits in African Americans**	*Mobiluncus curtisii/mulieris*	0.3816	1.00E-16	3.60E-15	4.001	303 (179)	58 (40)	0.0100
**Visit 1 in African Americans**	None	n/a	n/a	n/a	n/a	n/a	n/a	n/a
**All visits in non-African Americans**	None	n/a	n/a	n/a	n/a	n/a	n/a	n/a
**Nulliparous women**	None	n/a	n/a	n/a	n/a	n/a	n/a	n/a
**Multiparous women**	*Mobiluncus curtisii/mulieris*	0.3399	2.10E-13	1.45E-11	3.903	204 (130)	204 (130)	0.0125
**CST IVB women**	*Mobiluncus curtisii/mulieris*	0.4377	1.47E-04	8.52E-03	3.215	76 (47)	76 (47)	0.0610

Supplementary Table 5.

β-defensin-2 data and statistics in support of Figures 2, Supplementary Figures 5 and 6. p-values were estimated using a Bayesian 2-proportions binomial model with uniform prior implemented in *rstan* R package. Significant p-value are highlighted in bold. Significant p-values are highlighted in bold and yellow.

	TERM	sPTB	p-val
**All subjects**	4.329165	4.004237	**0.00061**

All subjects at V1 - Beta-defensin-2 log(quant)

	TERM (n)	sPTB (n)	p-val
**I**	4.16 (121)	3.68 (26)	0.030
**II**	4.41 (42)	4.18 (6)	0.500
**III**	4.46 (83)	4.02 (16)	**0.050**
**IV-A**	4.44 (87)	4.40 (32)	0.780
**IV-B**	4.35 (60)	3.80 (16)	**0.010**
**V**	4.12 (26)	3.65 (6)	0.120

	TERM (n)	sPTB (n)	p-val
**I**	14141.35 [109.95, 784211.12] (134)	4824.81 [35.20, 232820.89] (26)	**0.030**
**II**	26515.91 [925.41, 1379433.37] (44)	15100.31 [515.27, 63206.38] (6)	0.470
**III**	31417.29 [461.15, 1671281.50] (95)	10406.67 [542.13, 135216.48] (16)	**0.030**
**IV-A**	21651.44 [397.46, 758687.38] (65)	6270.79 [122.68, 58230.71] (16)	**0.010**
**IV-B**	29707.13 [14.05, 3395774.00] (109)	24844.25 [606.48, 2457369.50] (32)	0.640
**V**	13683.86 [1071.15, 549539.00] (29)	4477.76 [390.13, 23820.41] (6)	0.110

	Q1 sPTB/TERM (\%)	Q4 sPTB/TERM (\%)	p-val
**I**	10/37 (27.0)	5/36 (13.9)	0.094
**II**	1/12 (8.3)	3/12 (25.0)	0.183
**III**	8/25 (32.0)	3/24 (12.5)	0.073
**IV-A**	7/19 (36.8)	3/19 (15.8)	0.090
**IV-B**	6/30 (20.0)	10/29 (34.5)	0.116
**V**	3/8 (37.5)	2/8 (25.0)	0.315

	TERM	sPTB	p-val
**AA**	4.476802	4.100683	**0.00028**
**nonAA**	3.898671	3.691647	0.3271

AA at V1 - Beta-defensin-2 log(quant)

	TERM (n)	sPTB (n)	p-val
**I**	4.44 (64)	4.00 (15)	0.070
**II**	4.45 (32)	4.54 (3)	0.650
**III**	4.57 (70)	3.96 (11)	**0.050**
**IV-A**	4.33 (57)	3.80 (16)	**0.010**
**IV-B**	4.53 (80)	4.44 (26)	0.630
**V**	4.57 (9)	3.66 (5)	**0.030**

	TERM (n)	sPTB (n)	p-val
**I**	27813.29 [275.84, 784211.12] (64)	9971.59 [385.48, 232820.89] (15)	0.070
**II**	28202.74 [925.41, 1379433.37] (32)	34720.78 [21667.82, 63206.38] (3)	0.650
**III**	36788.78 [461.15, 1641368.62] (70)	9145.25 [542.13, 135216.48] (11)	**0.050**
**IV-A**	21482.21 [397.46, 758687.38] (57)	6270.79 [122.68, 58230.71] (16)	**0.010**
**IV-B**	33739.11 [14.05, 3187321.00] (80)	27538.12 [606.48, 2457369.50] (26)	0.630
**V**	37262.96 [6072.55, 141913.12] (9)	4529.19 [390.13, 23820.41] (5)	**0.030**

	Q1 sPTB/TERM (\%)	Q4 sPTB/TERM (\%)	p-val
**I**	6/20 (30.0)	3/20 (15.0)	0.147
**II**	0/9 (0.0)	0/9 (0.0)	0.498
**III**	6/21 (28.6)	0/20 (0.0)	**0.030**
**IV-A**	7/19 (36.8)	1/18 (5.6)	**0.030**
**IV-B**	6/27 (22.2)	4/27 (14.8)	0.255
**V**	3/4 (75.0)	0/4 (0.0)	0.069

Supplementary Table 6.

Statistical analysis in support of Figures 3 and Supplementary Figure 7. p-values were estimated using Bayesian binomial models using uniform prior for two proportions implemented in *rstan* R package. Significant p-values are highlighted in bold and yellow.

All subbjects at visit 1

sPTB counts

BD-2 tertiles	Lactobacillus tertiles		
	Lb T1	Lb T2	Lb T3
BD T3	6/18	1/17	1/20
BD T2	5/19	6/23	3/13
BD T1	6/18	3/15	9/22

Proportions of sPTB

BD-2 tertiles	Lactobacillus tertiles		
	Lb T1	Lb T2	Lb T3
BD T3	0.33	0.06	0.05
BD T2	0.26	0.26	0.23
BD T1	0.33	0.20	0.41

Relative risk of sPTB (proportion of sPTB/baseline proportion of sPTB (19.58%))

BD-2 tertiles	Lactobacillus tertiles		
	Lb T1	Lb T2	Lb T3
BD T3	1.70	0.30	0.26
BD T2	1.34	1.33	1.18
BD T1	1.70	1.02	2.09

p-value - Risk of sPTB compared to baseline risk (19.58%)

BD-2 tertiles	Lactobacillus tertiles		
	Lb T1	Lb T2	Lb T3
BD T3	0.067	0.107	0.069
BD T2	0.188	0.191	0.287
BD T1	0.067	0.390	**0.008**

African Americans at visit 1

sPTB counts

BD-2 tertiles	Lactobacillus tertiles		
	Lb T1	Lb T2	Lb T3
BD T3	2/11	2/15	1/18
BD T2	6/18	3/15	1/10
BD T1	6/15	4/13	7/16

Proportions of sPTB

BD-2 tertiles	Lactobacillus tertiles		
	Lb T1	Lb T2	Lb T3
BD T3	0.18	0.13	0.06
BD T2	0.33	0.20	0.10
BD T1	0.40	0.31	0.44

Relative risk of sPTB (proportion of sPTB/baseline proportion of sPTB (19.58%))

BD-2 tertiles	Lactobacillus tertiles		
	Lb T1	Lb T2	Lb T3
BD T3	0.93	0.68	0.28
BD T2	1.70	1.02	0.51
BD T1	2.04	1.57	2.23

p-value - Risk of sPTB compared to baseline risk (19.58%)

BD-2 tertiles	Lactobacillus tertiles		
	Lb T1	Lb T2	Lb T3
BD T3	0.431	0.370	0.096
BD T2	0.063	0.385	0.332
BD T1	**0.026**	0.122	**0.011**

All subjects at visit 1 - Two regions considered

sPTB counts

BD-2 tertiles	Lb T1	Lb T2	Lb T3
BD T3		11/68	
BD T2			
BD T1		29/97	

(Columns: Lactobacillus tertiles)

Proportions of sPTB

BD-2 tertiles	Lb T1	Lb T2	Lb T3
BD T3		0.16	
BD T2			
BD T1		0.30	

(Columns: Lactobacillus tertiles)

Relative risk of sPTB (proportion of sPTB/baseline proportion of sPTB (19.58%))

BD-2 tertiles	Lb T1	Lb T2	Lb T3
BD T3		0.83	
BD T2			
BD T1		1.53	

(Columns: Lactobacillus tertiles)

p-value - Risk of sPTB compared to baseline risk (19.58%)

BD-2 tertiles	Lb T1	Lb T2	Lb T3
BD T3		0.288	
BD T2			
BD T1		**0.002**	

(Columns: Lactobacillus tertiles)

African Americans at visit 1 - Two regions considered

sPTB counts

BD-2 tertiles	Lb T1	Lb T2	Lb T3
BD T3		6/54	
BD T2			
BD T1		26/77	

(Columns: Lactobacillus tertiles)

Proportions of sPTB

BD-2 tertiles	Lb T1	Lb T2	Lb T3
BD T3		0.11	
BD T2			
BD T1		0.34	

(Columns: Lactobacillus tertiles)

Relative risk of sPTB (proportion of sPTB/baseline proportion of sPTB (19.58%))

BD-2 tertiles	Lb T1	Lb T2	Lb T3
BD T3		0.57	
BD T2			
BD T1		1.72	

(Columns: Lactobacillus tertiles)

p-value - Risk of sPTB compared to baseline risk (19.58%)

BD-2 tertiles	Lb T1	Lb T2	Lb T3
BD T3		0.071	
BD T2			
BD T1		**0.035**	

(Columns: Lactobacillus tertiles)

Supplementary Data Files

Supplementary Data 1.
Taxonomic assignment tables of taxa relative abundance and associated metadata.

Supplementary Data 2.
Taxonomic assignment tables of taxa sequence counts and associated metadata.

Supplementary Data 3.
Taxonomic assignments for 14 positive control samples comprising of a mixture of samples with known compositions.
