# Supplementary material for: Cervicovaginal microbiota and local immune response modulate the risk of spontaneous preterm delivery
Source: Nat Commun. 2019 Mar 21;10:1305. doi: 10.1038/s41467-019-09285-9 (PMC6428888; doi:10.1038/s41467-019-09285-9)
Supplement: Supplementary file 3 — Description of Additional Supplementary Files [file 41467_2019_9285_MOESM3_ESM.docx]

**Description of Supplementary Files**

**File Name:** Supplementary Data 1.

**Description:** Taxonomic assignment tables of taxa relative abundance and associated metadata.

**File Name:** Supplementary Data 2.

**Description:** Taxonomic assignment tables of taxa sequence counts and associated metadata.

**File Name:** Supplementary Data 3.

**Description:** Taxonomic assignments for 14 positive control samples comprising of a mixture of samples with known compositions.
